# Genome-Wide Identification and Analysis of MicroRNAs Involved in Witches’-Broom Phytoplasma Response in *Ziziphus jujuba*

**DOI:** 10.1371/journal.pone.0166099

**Published:** 2016-11-08

**Authors:** Fenjuan Shao, Qian Zhang, Hongwei Liu, Shanfa Lu, Deyou Qiu

**Affiliations:** 1 State Key Laboratory of Tree Genetics and Breeding, Key Laboratory of Tree Breeding and Cultivation of State Forestry Administration, Research Institute of Forestry, Chinese Academy of Forestry, Beijing, China; 2 Institute of Medicinal Plant Development, Chinese Academy of Medical Sciences & Peking Union Medical College, Beijing, China; Dokuz Eylul Universitesi, TURKEY

## Abstract

MicroRNAs (miRNAs) play an important role in responding to biotic and abiotic stresses in plants. Jujube witches’-broom a phytoplasma disease of *Ziziphus jujuba* is prevalent in China and is a serious problem to the industry. However, the molecular mechanism of the disease is poorly understood. In this study, genome-wide identification and analysis of microRNAs in response to witches’-broom was performed. A total of 85 conserved miRNA unique sequences belonging to 32 miRNA families and 24 novel miRNA unique sequences, including their complementary miRNA* strands were identified from small RNA libraries derived from a uninfected and witches’-broom infected *Z*. *jujuba* plant. Differentially expressed miRNAs associated with Jujube witches’-broom disease were investigated between the two libraries, and 12 up-regulated miRNAs and 10 down- regulated miRNAs identified with more than 2 fold changes. Additionally, 40 target genes of 85 conserved miRNAs and 49 target genes of 24 novel miRNAs were predicted and their putative functions assigned. Using the modified 5’-RACE method, we confirmed that *SPL* and *MYB* were cleaved by miR156 and miR159, respectively. Our results provide insight into the molecular mechanisms of witches’-broom disease in *Z*. *jujuba*.

## Introduction

*Zizyphus jujuba* (common name Chinese Jujube) is an economically important fruit tree species in China, belonging to the family *Rhamnaceae* [1]. It is widely used in traditional Chinese medicine for at least 3,000 years, because its fruit contains high vitamin C content, abundant phenolic compounds, carbohydrate, minerals, cyclic AMP and other important nutrients [1–3]. Jujube witches’-broom (JWB) disease is prevalent in China and causes serious problems to the industry [4]. It is caused by phytoplasmas which are bacteria without cell walls that were first discovered in the phloem of plants in 1967 by Yoji Doi and co-workers [5]. Phytoplasmas are transmitted by phloem-sucking leafhoppers and Chinese Jujube plants infected with phytoplasmas display a variety of symptoms, such as small leaves, yellowing, witches’-broom, phyllody, stunting, sterile flowers and finally death after a few years of infection [6, 7]. Phytoplasmas are very destructive agricultural pathogens, and have devastating effects on over 1000 plant species worldwide [8, 9].

A previous study of Mexican lime trees infected with phytoplasma identified several candidate genes and proteins that might be involved in the interaction of Mexican lime trees with the phytoplasma [10, 11]. Although some progress has been made in understanding the regulation that is involved in plant-phytoplasma interactions [12], the molecular mechanisms involved in the JWB disease and the symptoms are poorly understood [13].

In recent years, many studies have shown that small RNAs (sRNAs) have numerous roles in the development of plants, defense against viruses and transposons, chromatin modifications, responses to biotic and abiotic stresses etc. In plants, microRNAs (miRNAs) and small interfering RNAs (siRNAs) are two major classes of small RNAs [14]. miRNAs are produced from the primary miRNA transcripts with internal stem-loop structures, whereas siRNAs are derived from dsRNAs transcripts. To regulate gene expression, the generated sRNA are loaded into RNA-induced silencing complexes (RISCs) to guide and interact with homologous RNA or DNA molecules for direct RNA cleavage, translational repression or DNA methylation [15].

High-throughput sequencing provides a comprehensive means of identifying and studying the expression of small RNAs. miRNAs play an important role in disease resistance in plants [16–20], for example, a total of 87 differentially regulated miRNAs have been identified to be responsive to fungal stress in wheat [20]. However, to our best knowledge, there is no report on miRNAs associated with JWB in *Z*. *jujuba*. Understanding the molecular mechanisms of witches’-broom disease associated with miRNAs is potentially important for developing efficient methods to control the disease. With the aim of identifying miRNAs involved in JWB disease, we constructed two small RNAs libraries from the sprig leaves of uninfected wild type (ZZN) *Z*. *jujuba* plants and plants with JWB disease (ZZD). miRNAs and their targets were identified from both small RNA libraries and differentially expressed miRNAs associated with JWB disease were determined. Our results provide insight into the molecular mechanisms of JWB disease in *Z*. *jujuba*.

## Materials and Methods

### Plant materials

The *Z*. *jujuba* wild type (ZZN) and the infected plant (ZZD) with witches’-broom disease used in this experiment were grown in Beijing Olympic Park (116°40′7.43"E, 39°99′9.45"N). Sprig of *Z*. *jujuba* leaves were collected from 10-year-old plants with the permission granted by the administrative department of Olympic Park. For each sample, materials from three plants were pooled and stored in liquid nitrogen until use.

### Small RNA library construction

Total RNAs were extracted from the wild type (ZZN) and the infected plant (ZZD) using Trizol RNA extraction kit (Life Technology, Beijing) according to the manufacturer’s instruction. Two small RNA samples were sequenced by Novogene (China) using Illumina HiSeq2500 system, and the raw reads generated by Illumina sequencing were submitted to the SRA database, Accession No. SRP090598.

### Bioinformatics analysis of sequencing data

After removing the adapters and low-quantity sequences from the raw reads, the 18–30 nt clean reads were compared with Rfam database and the NCBI nucleotide database to removed the rRNA, tRNA, snRNA and snoRNA for further analyses. The remaining sequences in the ZZN and ZZD libraries at least ten reads were searched against miRBase 21.0 with a maximum of three mismatches allowed [21] to identify conserved miRNAs in *Z*. *jujuba*, and then the resulting sequences were screened for the presence of the characteristic hairpin structures using the program RNAfold [22]. The software Mireap (https://sourceforge.net/projects/mireap/) was used to predict novel miRNAs, which could be mapped to the *Z*. *jujuba* genome. The resulting secondary structures were then manually checked. Criteria described by Meyers et al were applied to annotate the novel miRNAs [23]. The reads of small RNAs were normalized to one million by the total number of small RNAs in each library for comparing the differential expression levels of the miRNAs in the ZZN and ZZD libraries.

### Target gene prediction for miRNAs

Target genes prediction of the known and novel miRNAs was performed against assembled *Z*. *jujuba* unigenes using psRNATarget [24]. The maximum expectations of 3 and the target accessibility-allowed maximum energy to unpair the target site of 50 were applied. The functions of targets were annotated by blast analysis against the Nr protein database [25] using default parameters.

### Quantitative RT-PCR

MicroRNAs expression levels were quantified using Poly (A) Tailing method, following the previously reported procedures [26]. In brief, the 1μg DNaseI treated total RNA was polyadenylated by Poly (A) polymerase at 37°C for 1 h in a 20-μL reaction mixture following the manufacturer’s directions for the Poly (A) Tailing Kit (Ambion). The all RNAs were reverse-transcribed with 200 U SuperScript™ III Reverse Transcriptase (Invitrogen) using poly (T) adapters. Zj5.8S rRNA was used as a control as previously described [27]. Gene-specific primers were listed in S1 Table.

### Validation of target cleavage sites by 5’-RLM-RACE

The 5’-RLM-RACE experiments were carried out using the modified RNA ligase-mediated rapid amplification of 5’ cDNAs method as described [28,29], PCRs were carried out on mRNA isolated from *Z*. *jujuba* infected with witches’-broom disease using the GeneRacer 5’ primer and the nesting gene-specific primers (S2 Table). Nested PCRs were performed using the GeneRacer 5’ nested primer and the nested gene-specific primers (S2 Table).

## Results

### Overview of the small RNA sequences

**Fig 1:**
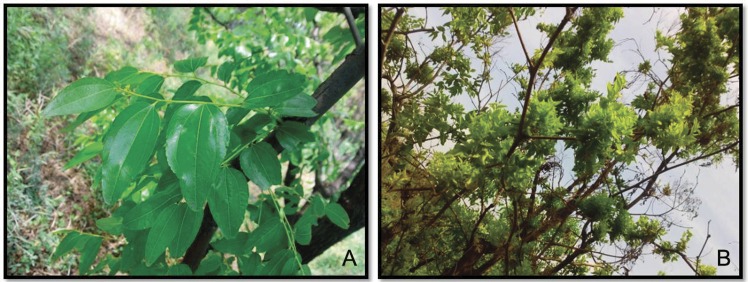
The *Ziziphus jujuba* wild type (ZZN) and the infected plant (ZZD) with witches broom disease in the field. A. ZZN; B. ZZD.

**Fig 2:**
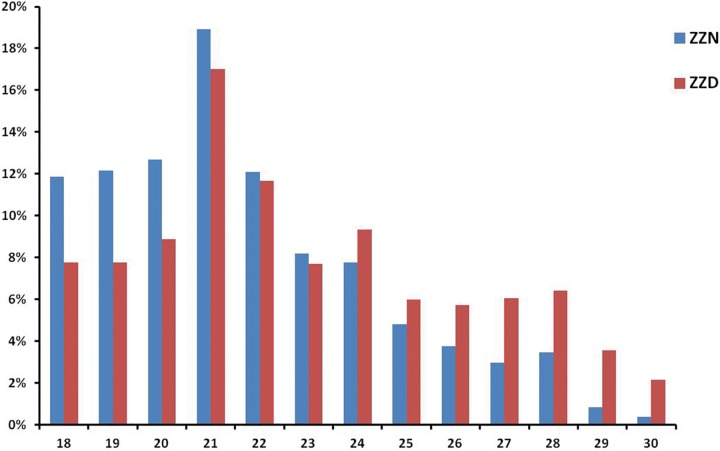
The size distribution of the small RNAs in ZZN and ZZD libraries.

Two small RNA libraries were constructed from the sprig leaves of *Z*. *jujuba* wild type (ZZN) and the infected plant (ZZD) with witches’-broom disease (Fig 1). Using the Illumina sequencing technology, a total of 14,171,805 and 11,483,382 raw reads were generated for ZZN and ZZD, respectively. After removing contaminant reads and filtering out the adapter sequences, 13,729,929 and 11,150,259 clean reads with lengths of 18 to 30nt were obtained for ZZN and ZZD, respectively (Table 1). In both libraries, most of total sRNA reads were 18- 24nt in size (Fig 2). The most abundant small RNAs in the both libraries were 21 nt sRNA, which were approximately 18.91% (ZZN) and 17.01% (ZZD) of the total sequence reads in ZZN and ZZD libraries, respectively. Whereas, the abundance of 24-nt sRNAs in ZZD library (9.34%) were higher than in ZZN library (7.78%). The 24-nt sRNAs were mainly comprised of siRNAs, suggesting it may play an important role in the regulation of the response to the phytoplasma infection in plants.

**Table 1:** Statistics of small RNA sequences from ZZN and ZZD libraries.

Library	total reads	low quality	clean reads	Unique reads
ZZD	11483382	9219	11150259	1795277
ZZN	14171805	9737	13729929	1249861

### Identification of conserved miRNAs in *Z*. *jujuba*

To identify the conserved miRNAs in *Z*. *jujuba*, the unique sequences with at least 10 reads in the both sRNA libraries were mapped to the *Z*. *jujuba* genome [30] with no more than 2 mismatches. All the mapped sRNA was aligned with known mature plant miRNAs in miRBase 21.0 by UEA small RNA tools [31] and a maximum of three mismatches were allowed. As a result, we identified 85 unique sequences, belonging to 32 families in the both sRNA libraries generated by Illumina sequencing (Table 2).

**Table 2:** The conserved miRNAs of *Z*. *jujuba*.

miRNA family	members	sequences	reads	
			ZZN	ZZD
miR1515	miR1515	UCAUUUUUGCGUGCAAUGAUCC	16	5
miR156	miR156a	UUGACAGAAGAGAGUGAGCAC	32	472
	miR156b	UGACAGAAGAGAGUGAGCACU	25	225
	miR156c	UUGACAGAAGAUAGAGAGCAC	18	295
	miR156d	UGACAGAAGAGAGUGAGCAC	22	1170
	miR156e	UUGACAGAAGAUAGAGAGCA	4	16
	miR156f	UUGACAGAAGAGAGAGAGCAC	30	40
	miR156h	UGACAGAAGAGAGUGAGCACA	3	28
miR159	miR159a	UUGGAUUGAAGGGAGCUCCA	11	1
	miR159b	UUUGGAUUGAAGGGAGCUCU	68830	26691
	miR159c	UUUGGAUUGAAGGGAGCUCUA	72287	40499
	miR159d	UUGGAUUGAAGGGAGCUCUA	1545	724
	miR159e	CUUGGAUUGAAGGGAGCUCC	315	2255
miR160	miR160a	UGCCUGGCUCCCUGUAUGCCA	85	102
	miR160b	UGCCUGGCUCCCUGAAUGCC	37	18
	miR160c	UGCCUGGCUCCCUGUAUGCC	63	61
	miR160d	UGCCUGGCUCCCUGAAUGCCA	78	53
miR162	miR162a	UCGAUAAACCUCUGCAUCCAG	6233	3051
	miR162b	UCGAUAAACCUCUGCAUCCA	26	9
miR164	miR164a	UGGAGAAGCAGGGCACGUGC	44	15
	miR164b	UGGAGAAGCAGGGCACGUGCA	135	138
miR166	miR166a	UCGGACCAGGCUUCAUUCCCCC	998	307
	miR166b	GGAAUGUUGUCUGGCUCGAGG	30	22
	miR166c	UCGGACCAGGCUUCAUUCCCC	54342	23732
	miR166d	UCGGACCAGGCUUCAUUCCC	340	254
	miR166e	UCGGACCAGGCUUCAUUCCU	274	195
	miR166f	UCGGACCAGGCUUCAUUCCUC	345	271
	miR166g	UCUCGGACCAGGCUUCAUUCC	58	36
miR167	miR167a	UGAAGCUGCCAGCAUGAUCU	425	171
	miR167b	UGAAGCUGCCAGCAUGAUCUUA	911	348
	miR167c	UGAAGCUGCCAGCAUGAUCUG	15165	6839
	miR167d	UGAAGCUGCCAGCAUGAUCUGG	518	291
	miR167e	UGAAGCUGCCAGCAUGAUCUAA	22	12
	miR167f	UGAAGCUGCCAGCAUGAUCUA	2577	993
	miR167g	UGAAGCUGCCAGCAUGAUCUU	2768	800
	miR167h	UGAAGCUGCCAGCAUGAUCUGA	13454	7103
miR168	miR168a	UCGCUUGGUGCAGGUCGGGAA	1090	582
	miR168b	CCCGCCUUGCAUCAACUGAAU	78	36
	miR168c	UCGCUUGGUGCAGGUCGGGA	86	44
miR170	miR170	UAUUGGCCUGGUUCACUCAGA	141	420
miR171	miR171a	UUGAGCCGCGCCAAUAUCACU	11	35
	miR171b	UGAUUGAGCCGUGCCAAUAUC	46	92
miR172	miR172	AGAAUCUUGAUGAUGCUGCAU	259	5
miR2111	miR2111	UAAUCUGCAUCCUGAGGUUUA	21	1
miR2950	miR2950	UUCCAUCUCUUGCACACUGGA	189	11
miR319	miR319a	UUGGACUGAAGGGAGCUCCCU	46	346
	miR319b	CUUGGACUGAAGGGAGCUCCC	37	47
	miR319c	UUUGGACUGAAGGGAGCUCCU	29	18
	miR319d	AUUGGACUGAAGGGAGCUCC	55	56
	miR319e	UUGGACUGAAGGGAGCUCCC	392	645
	miR319f	CUUGGACUGAAGGGAGCUCCU	83	58
	miR319g	UUGGACUGAAGGGAGCUCCU	438	479
miR384	miR384	UUGGCAUUCUGUCCACCUCC	52	82
miR390	miR390	CGCUAUCCAUCCUGAGUUUCA	7	12
miR391	miR391a	UACGCAGGAGAGAUGACGCCG	1095	289
	miR391b	UACGCAGGAGAGAUGACGCC	56	29
miR394	miR394	UUGGCAUUCUGUCCACCUCC	52	82
miR395	miR395a	UGAAGUGUUUGGGGGAACUCC	6	29
	miR395b	CUGAAGUGUUUGGGGGGACUC	22	75
miR396	miR396a	CUCAAGAAAGCUGUGGGAGA	63	47
	miR396b	UUCAAUAAAGCUGUGGGAAG	61	32
	miR396c	CACAGCUUUCUUGAACUUUCU	37	20
	miR396d	UUCCACAGCUUUCUUGAACUG	14559	6996
	miR396e	UUCCACAGCUUUCUUGAACUU	39754	31938
	miR396f	UUCCACAGCUUUCUUGAACU	2692	1631
	miR396g	GUUCAAUAAAGCUGUGGGAAG	36	33
	miR396h	UCCACAGCUUUCUUGAACUU	19	26
miR397	miR397	UCAUUGAGUGCAGCGUUGAUG	53	23
miR398	miR398a	UGUGUUCUCAGGUCGCCCCU	319	67
	miR398b	UGUGUUCUCAGGUCGCCCCUG	11405	4867
	miR398c	UGUGUUCUCAGGUCACCCCUU	607	148
miR399	miR399	UGCCAAAGGAGAGUUGCCCUG	33	2
miR403	miR403	UUAGAUUCACGCACAAACUCG	204	164
miR408	miR408a	UGCACUGCCUCUUCCCUGGCU	1791	671
	miR408b	AUGCACUGCCUCUUCCCUGGC	872	241
	miR408c	UGCACUGCCUCUUCCCUGGC	814	257
miR477	miR477	ACUCUCCCUCAAGGGCUUCU	78	4
miR482	miR482a	UGCCUAUUCCUCCCAUGCCAA	29	29
	miR482b	GGAAUGGGCUGUUUGGGAUG	11	14
miR529	miR529a	AGAAGAGAGAGAGUACAGCUU	1526	3435
	miR529b	AGAAGAGAGAGAGUACAGCU	70	128
miR530	miR530	UCUGCAUUUGCACCUGCACCU	23	17
miR6478	miR6478	CCGACCUUAGCUCAGUUGGU	837	289
miR858	miR858a	UUCGUUGUCUGUUCGACCUUG	516	124
	miR858b	UUCGUUGUCUGUUCGACCUGA	85	12

**Fig 3:**
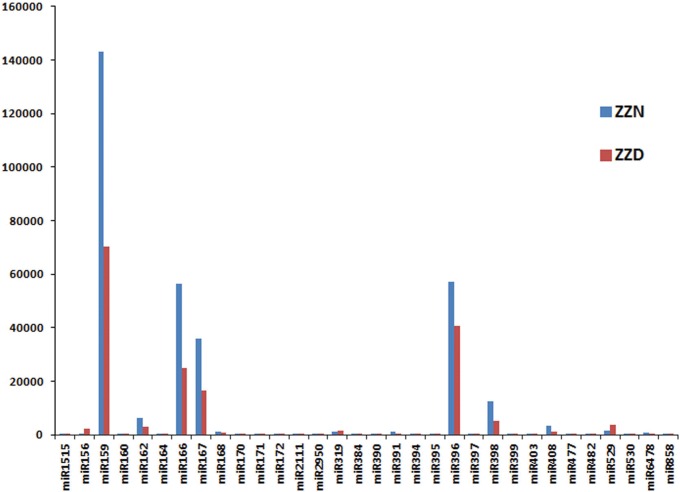
Number of reads for the conserved miRNA families.

Among the 32 identified miRNA families, a total of 18 miRNA families contained several members, and seven families including miR156, miR159, miR160, miR166, miR167, miR319 and miR396, had at least four members; 13 miRNA families, namely miR170, miR172, miR384, miR390, miR397, miR399, miR403, miR477, miR530, miR1515, miR2111, and miR2950 and miR6478, had only one member. Of these families, miR159 was the most abundant, with 142988 (ZZN) and 70170 (ZZD) reads accounting for 44.3% and 40.8% of all conserved miRNAs in both libraries, respectively (Fig 3). The second most abundant miRNA family is miR396, with 57221 (ZZN) and 40723 (ZZD) reads accounting for 17.7% and 23.7% of all conserved miRNAs in both libraries. The third most abundant miRNA family was miR166 and miR167. The other conserved miRNA families showed less abundance and each had less than 0.2% of all conserved miRNA reads. This result is significantly different with other plants, suggesting differential expression of miRNAs in *Z*. *jujuba* and indicating there is significant diversity of miRNA expression in different plant species.

### Identification of novel miRNAs in *Z*. *jujuba*

We used criteria described by Meyers et al [23] to identify novel miRNAs. As a result, we identified 24 novel miRNA sequences with a characteristic stem-loop precursor (Table 3). These novel miRNAs were given names designated as ‘zju-miRn plus number’. Among these novel miRNAs, 14 miRNA had miRNA* sequences, the other 10 had no miRNA* sequences. The length of the predicted novel miRNA precursors varied from 60 to 364 nt, and the average minimum free energy (MFE) value varied from -22 to -111.8 kcal/mol. Most of the novel miRNAs were 21 nt long and had uracil (U) as their first nucleotide. The structures of 24 novel miRNA precursors are shown in S1 Fig. Most of them showed differential expression in both libraries. For instance, the mature miRNA reads varied from 0 to 12561, and the miRNA* reads varied from 0 to 8024. The reads for most of these novel miRNA*s were less than their corresponding mature miRNAs except zju-miRn15* in both libraries. To investigate whether these 24 novel miRNA sequences were conserved across plant species, we used them as query sequences to search against the plant mature miRNAs in miRBase 21.0 by Blastn [32]. The results showed that no perfect matches were found, suggesting that these novel miRNA sequences were not broadly conserved in plants.

**Table 3:** The novel miRNAs identified in *Z*. *jujuba*.

Name	Sequence(5'-3')	Location	Length of	MFE	miRNA reads		miRNA* reads	
			precursors(nt)	(kcal/mol)	ZZN	ZZD	ZZN	ZZD
zju-miRn1	ACTGTCGCGGGAGAGATGGCTC	gi|699272003|gb|JREP01027427.1|:2299–2204	96	-36.4	46	11	2	0
zju-miRn2	CGGTGTGCAAGAAATGGAATA	gi|699280098|gb|JREP01019335.1|:30687–30620	68	-30.05	16	1	1	0
zju-miRn3	AATTCCGGCGATGGCGACTGC	gi|699293024|gb|JREP01006409.1|:14748–14812	65	-37.1	20	4	3	0
zju-miRn4	TTTCTACGTCCGGCGCAACATGTT	gi|699277485|gb|JREP01021948.1|:39003–39091	89	-31.34	7	4	0	1
zju-miRn5	TGGATCTTGTTCGATGGCACT	gi|699281215|gb|JREP01018218.1|:26882–26968	107	-44	12561	6701	507	235
zju-miRn6	TGCCTGGCTCCCTGTATGCC	gi|699278012|gb|JREP01021421.1|:2374–2457	84	-47.5	1	1	0	0
zju-miRn7	TATGATGCGGACGGTCCTCAT	gi|699270789|gb|JREP01028641.1|:12613–12776	164	-73.2	8	10	0	0
zju-miRn8	TCGTGTTCGGGTTAGGCATTT	gi|699270875|gb|JREP01028555.1|:4772–4848	77	-29.6	54	10	0	10
zju-miRn9	TCCTCAGTAGCTCAGTGGTA	gi|699273880|gb|JREP01025552.1|:9956–10142	187	-59.2	77	55	0	0
zju-miRn10	TGGGGAATGCCATGTAGACTTG	gi|699287923|gb|JREP01011510.1|:31269–31347	79	-30.4	10	3	0	0
zju-miRn11	ACAGGCGGTGGATCAAATATGAAT	gi|699283153|gb|JREP01016280.1|:34764–34705	60	-34	623	388	41	23
zju-miRn12	TTACAAGGTCGGTTGCATGGC	gi|699274146|gb|JREP01025287.1|:19052–19415	364	-111.8	8	10	0	0
zju-miRn13	TGTTCCTGCACAGATTCTCCC	gi|699278034|gb|JREP01021399.1|:11–92	82	-43.8	92	55	42	31
zju-miRn14	TGGATCTTGTTCGACAGCACT	gi|699291620|gb|JREP01007813.1|:1267–1375	109	-45	473	986	0	12
zju-miRn15	TTGGCGTGAGAGACTTGGTAG	gi|699297613|gb|JREP01001820.1|:3185–3308	124	-42.2	47	84	5782	8024
zju-miRn16	TTAGTGATCCTCCGGAAGATC	gi|699288201|gb|JREP01011232.1|:20386–20161	226	-73.33	490	55	76	3
zju-miRn17	TTAGATGGCCATCAACGAACA	gi|699277419|gb|JREP01022014.1|:763–685	80	-34.7	222	49	24	3
zju-miRn18	TATGGTGCGGACGGTCTTCAT	gi|699290074|gb|JREP01009359.1|:3643–3786	145	-78.1	20	12	11	0
zju-miRn19	TATGGTGCGGACGATCCTCAT	gi|699274854|gb|JREP01024579.1|:3620–3784	165	-70.3	30	18	0	0
zju-miRn21	TCAAATGATGAGTATGGATT	gi|699276408|gb|JREP01023025.1|:4365–4715	351	-79.7	49	86	0	0
zju-miRn22	TTTGCTTTGGAATCATTCTG	gi|699280203|gb|JREP01019230.1|:26219–26333	115	-31.1	15	29	0	0
zju-miRn23	TTTTCACCTCTTCTGGACGGG	gi|699280638|gb|JREP01018795.1|:9272–9354	83	-22	4	12	0	0
zju-miRn24	TCCAGGAAGCTGTTTCTCAT	gi|699285304|gb|JREP01014129.1|:6565–6729	165	-35.5	0	12	0	0
zju-miRn25	TCTATGGTGAGGACGGTCCA	gi|699292052|gb|JREP01007381.1|:20187–20358	172	-77.8	0	49	15	0

### Targets of conserved and novel miRNAs in *Z*. *jujuba*

To better understand the functions of identified miRNAs, we performed a target search of identified miRNAs against the jujube transcriptome unigenes using psRNATarget with penalty scores of 2.5 [24]. As a result, we identified a total of 150 unigenes from 65,534 assembled *Z*. *jujuba* unigenes to be targets of 32 conserved miRNA families (Table 4). Since the direction of unigenes could not be determined, we manually checked the direction of the predicted targets. Finally, we have identified 40 targets of 32 conserved miRNA families with penalty scores of 2.5.

**Table 4:** The predicted conserved miRNAs targets of *Z*. *jujuba*.

miRNA	Target gene	Target gene annotation
miR1515	comp41442_c0_seq1	glycoside hydrolase
miR156a	comp40466_c0_seq3	PREDICTED: squamosa promoter-binding-like protein 12-like
miR156b	comp36565_c2_seq1	PREDICTED: squamosa promoter-binding-like protein 13A-like
miR156c	comp36565_c2_seq1	PREDICTED: squamosa promoter-binding-like protein 13A-like
miR156f	comp42825_c0_seq1	squamosa promoter binding-like protein
miR159a	comp34167_c1_seq3	PREDICTED: transcription factor GAMYB-like
miR160a	comp39955_c2_seq9	Auxin response factor
miR164a	comp33975_c1_seq1	NAC domain protein NAC1
miR164a	comp33455_c0_seq1	PREDICTED: NAC domain-containing protein 100-like
miR166a	comp37537_c2_seq1	PREDICTED: homeobox-leucine zipper protein REVOLUTA
miR171b	comp16216_c0_seq1	PREDICTED: cytokinin hydroxylase
miR171b	comp41445_c7_seq1	PREDICTED: scarecrow-like protein 6-like
miR172	comp30896_c1_seq1	PREDICTED: ethylene-responsive transcription factor RAP2-7-like
miR2111a	comp33728_c0_seq1	Cell division protein ftsZ, putative
miR2950a	comp38329_c0_seq1	PREDICTED: DEAD-box ATP-dependent RNA helicase 46-like
miR319b	comp28710_c0_seq1	PREDICTED: probable E3 ubiquitin-protein ligase ARI7-like
miR319d	comp34167_c1_seq3	PREDICTED: transcription factor GAMYB-like
miR394	comp32572_c0_seq1	zinc finger, C2H2, LYAR-type protein
miR395a	comp38415_c5_seq4	nucleic acid binding protein, putative
miR395b	comp13922_c0_seq1	PREDICTED: L-gulonolactone oxidase-like
miR395b	comp28089_c0_seq1	PREDICTED: probable serine/threonine-protein kinase Cx32
miR396a	comp11572_c0_seq1	DNA-dependent RNA polymerase II second largest subunit
miR396a	comp20253_c0_seq1	putative rna-dependent rna polymerase protein
miR396b	comp10489_c0_seq1	PREDICTED: DUF246 domain-containing protein
miR396b	comp15448_c0_seq1	ribosomal protein L13A
miR396c	comp40813_c0_seq2	PREDICTED: putative calcium-transporting ATPase 13
miR396c	comp41603_c3_seq4	PREDICTED: ribonuclease 3-like protein 2-like
miR396d	comp33509_c0_seq1	Growth-regulating factor
miR396d	comp37447_c0_seq15	PREDICTED: growth-regulating factor 1-like
miR396d	comp18240_c0_seq1	PREDICTED: growth-regulating factor 8-like
miR397	comp36338_c0_seq1	laccase 1b
miR398c	comp35319_c0_seq1	PREDICTED: pollen-specific protein SF3-like
miR399	comp38254_c1_seq1	PREDICTED: probable ubiquitin-conjugating enzyme E2 24-like
miR477	comp57357_c0_seq1	DELLA protein GAI1, putative
miR477	comp44262_c0_seq1	PREDICTED: germin-like protein subfamily 1 member 1-like
miR482a	comp40948_c0_seq8	NBS type disease resistance protein
miR529a	comp42121_c0_seq1	Disease resistance protein RPM1
miR530	comp39924_c5_seq4	acetyl-CoA carboxylase BCCP subunit
miR858a	comp23909_c0_seq1	GHMYB10
miR858b	comp47693_c0_seq1	NBS-LRR disease-resistance protein scn3r1

The putative functions of the predicted target genes were diverse, most of the target genes were transcription factors, disease resistant genes or the key enzyme genes involved in development, disease resistance or metabolism. Most targets of conserved miRNAs in *Z*. *jujuba* were the same to those reported miRNAs in other plant species, such as squamosa promoter binding-like protein genes targeted by miR156, transcription factor GAMYB-like gene regulated by miR159, auxin response factor gene regulated by miR160, NAC-domain protein gene, homeobox-leucine zipper protein gene, AP2 domain-containing protein gene, growth regulating factor gene, laccase gene and MYB gene targeted by miR164, miR166, miR172, miR396, miR397, and miR858, respectively (Table 4).

Using the same approach, we predicted 49 targets for 24 novel miRNA genes (Table 5). The number of predicted targets varied from 1 to 4 per miRNA. Many of the predicted targets are associated with metabolism, signal transduction and development. We found that four ubiquitin carboxyl-terminal hydrolase 5-like genes were the targets of miRn7, miRn18, miRn19 and miRn24, respectively, and two serine/threonine protein kinase genes were the targets of miRn5 and miRn14. This phenomenon that one gene can be targeted by more than one miRNAs also have been found in other plant species [27].

**Table 5:** The predicted novel miRNAs targets of *Z*. *jujuba*.

miRNA	Target gene	Target gene annotation
zju-miRn1	comp18278_c0_seq1	adenylate cyclase
zju-miRn2	comp45157_c0_seq1	PREDICTED: BAG family molecular chaperone regulator 3-like
zju-miRn2	comp37427_c0_seq12	PREDICTED: uncharacterized lipoprotein syc1174_c-like
zju-miRn2	comp40764_c4_seq145	nucleic acid binding protein, putative
zju-miRn2	comp40676_c1_seq5	cellulose synthase
zju-miRn3	comp38877_c0_seq7	PREDICTED: methylmalonate-semialdehyde dehydrogenase
zju-miRn4	comp32382_c1_seq1	Tigger transposable element-derived protein 6
zju-miRn4	comp40514_c0_seq2	AP-1 complex subunit gamma-2, putative
zju-miRn4	comp31728_c0_seq1	PREDICTED: major allergen Pru ar 1-like
zju-miRn5	comp41887_c1_seq80	serine/threonine protein kinase
zju-miRn5	comp35892_c1_seq2	PREDICTED: ethylene-responsive transcription factor ERF015-like
zju-miRn6	comp33310_c2_seq1	putative auxin response factor ARF16
zju-miRn6	comp39955_c2_seq9	Auxin response factor, putative
zju-miRn7	comp41963_c4_seq2	PREDICTED: ubiquitin carboxyl-terminal hydrolase 5-like
zju-miRn8	comp35801_c1_seq2	PREDICTED: uncharacterized LOC101216743
zju-miRn9	comp40169_c0_seq1	sieve element occlusion
zju-miRn9	comp41105_c1_seq1	family 5 glycoside hydrolase
zju-miRn10	comp27249_c0_seq1	PREDICTED: xyloglucan galactosyltransferase KATAMARI1-like
zju-miRn10	comp41841_c2_seq3	PREDICTED: leucine-rich repeat receptor protein kinase EXS-like
zju-miRn11	comp20342_c0_seq1	PREDICTED: DNA helicase INO80-like
zju-miRn11	comp42075_c1_seq3	PREDICTED: beta-galactosidase-like
zju-miRn12	comp37762_c0_seq1	clathrin, heavy polypeptide
zju-miRn13	comp33278_c0_seq4	sentrin/sumo-specific protease, putative
zju-miRn14	comp41887_c1_seq80	serine/threonine protein kinase
zju-miRn14	comp32418_c0_seq3	PREDICTED: tetratricopeptide repeat protein 27 homolog
zju-miRn15	comp16621_c0_seq1	short chain dehydrogenase/reductase
zju-miRn15	comp35884_c2_seq3	glutathione peroxidase
zju-miRn15	comp52942_c0_seq1	ubiquitination network signaling protein
zju-miRn15	comp57387_c0_seq1	GDP-mannose 4,6-dehydratase
zju-miRn16	comp40793_c0_seq2	80 kD MCM3-associated protein, putative
zju-miRn17	comp37942_c0_seq1	PREDICTED: SPX domain-containing membrane protein At4g22990
zju-miRn17	comp40255_c1_seq15	PREDICTED: dual specificity protein kinase pyk1-like
zju-miRn18	comp41963_c4_seq2	PREDICTED: ubiquitin carboxyl-terminal hydrolase 5-like
zju-miRn19	comp41963_c4_seq2	PREDICTED: ubiquitin carboxyl-terminal hydrolase 5-like
zju-miRn20	comp34156_c0_seq2	WRKY transcription factor, putative
zju-miRn20	comp37782_c0_seq8	PREDICTED: E3 ubiquitin-protein ligase XBAT33
zju-miRn20	comp39627_c0_seq3	PREDICTED: mitochondrial carnitine/acylcarnitine carrier-like protein-like
zju-miRn20	comp41133_c0_seq1	PREDICTED: pyruvate, phosphate dikinase, chloroplastic-like
zju-miRn21	comp32995_c0_seq1	NIPA2 protein
zju-miRn22	comp35463_c0_seq1	PREDICTED: DNL-type zinc finger protein-like
zju-miRn22	comp37131_c0_seq3	PREDICTED: O-glucosyltransferase rumi-like
zju-miRn22	comp55704_c0_seq1	S-adenosylmethionine-dependent methyltransferase, putative
zju-miRn23	comp37189_c0_seq1	trehalose-6-phosphate synthase, putative
zju-miRn23	comp37224_c0_seq11	PREDICTED: ubiquitin carboxyl-terminal hydrolase 22-like
zju-miRn23	comp41601_c5_seq8	PREDICTED: poly(U)-binding-splicing factor PUF60-B-like isoform 1
zju-miRn23	comp41632_c0_seq4	Pre-mRNA-processing factor
zju-miRn24	comp36352_c0_seq1	PREDICTED: probable galactinol—sucrose galactosyltransferase 2-like
zju-miRn24	comp37648_c0_seq1	PREDICTED: tyrosine—tRNA ligase-like
zju-miRn24	comp41963_c4_seq2	PREDICTED: ubiquitin carboxyl-terminal hydrolase 5-like

### Differentially expressed miRNAs in response to witches’-broom phytoplasma

To further identify the functions of miRNAs in *Z*. *jujuba* involved in response to phytoplasma infection, we normalized the expression levels of miRNAs and compared the expression levels of the miRNAs in the ZZN and ZZD libraries. As a result, we identified 85 conserved miRNA sequences and 24 novel miRNA sequences, 12 miRNA sequences were up-regulated more than 2 fold in the infected sprig leaves, including miR156a, miR156b, miR156c, miR156d, miR156e, miR156h, miR159e, miR319a, miR395a, miR395b, zju-miRn23 and zju-miRn24. Conversely, 10 miRNA sequences were down- regulated more than 2 fold in the infected plant (S3 Table), including miR159a, miR172, miR2111, miR2950, miR399, miR477, miR858b, zju-miRn2, zju-miRn8 and zju-miRn16. Among them, the most abundant up-regulated miRNAs were miR156a, but the most abundant down- regulated miRNA was miR172. Interestingly, in miR159 family, miR159e was up-regulated in the infected sprig leaves, whereas miR159a was down-regulated. In addition, most conserved and novel miRNAs were detected in both libraries, except zju-miRn23 and zju-miRn24 which were only detected in the ZZD library. This suggests that these two novel miRNAs may be related to response to phytoplasma infection.

**Fig 4:**
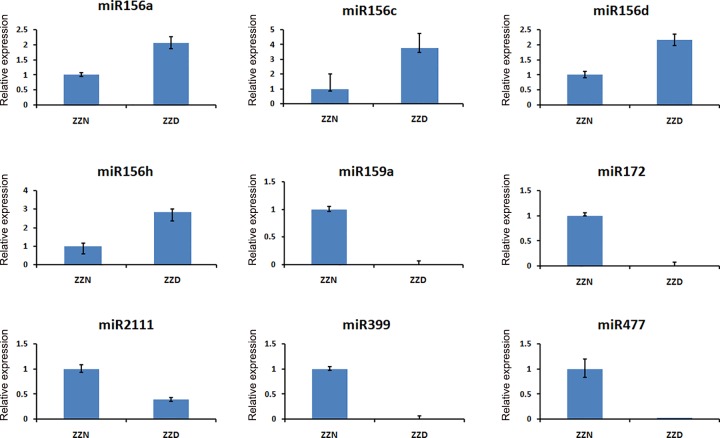
qRT-PCR validation of the differentially expressed miRNAs. Fold changes of the differentially expressed miRNAs are shown. miRNAs were analyzed using the poly(T) adaptor RT-PCR method. The levels in ZZN were arbitrarily set to 1. Error bars represent the standard deviations of three technical PCR replicates.

To validate the sequencing data and confirm the differential expression of the miRNAs, we performed poly(A) qRT-PCR on 9 miRNAs (miR156a, miR156c, miR156d, miR156h, miR159a, miR172, miR2111, miR399 and miR477) which were up-regulated or down- regulated more than 2 fold in the infected sprig leaves. The results revealed that miR156a, miR156c, miR156h and miR156d were up-regulated in the infected sprig leaves, whereas miR159a, miR172, miR2111, miR399 and miR477 were down- regulated in the infected sprig leaves. The results indicated that these 9 miRNAs had the same expression patterns compared with the sequencing data (Fig 4). These results imply that the phytoplasma responsive miRNAs in the regulation of biological processes involved in witches’-broom diseases.

### Experimental validation of *Z*. *jujuba* miRNAs targets

**Fig 5:**
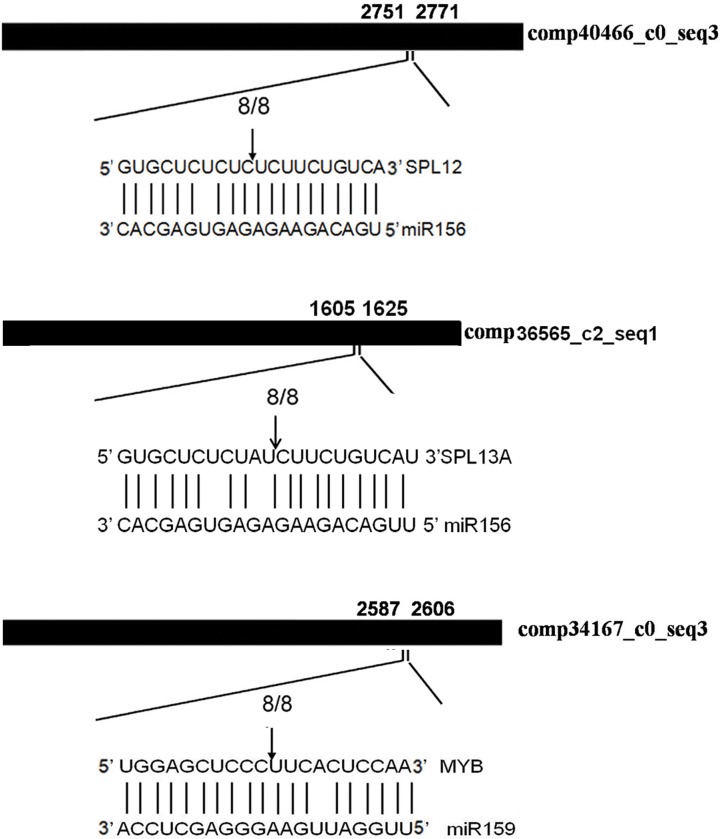
Experimental validation of the miRNA targets. Cleavage sites were determined by the modified 5’RNA ligase-mediated RACE. Heavy black lines represent unigenes. miRNA complementary sites with the nucleotide positions of SPL and MYB cDNAs are indicated. Vertical arrows indicate the 5’ termini of miRNA-guided cleavage products, as identified by 5’-RACE, with the frequency of clones shown.

To confirm whether the five differentially expressed miRNAs (miR156, miR159, miR172, miR2111 and miR477) could cleave the predicted targets, we isolated RNAs from the sprig leaves of *Z*. *jujuba* wild type (ZZN) and the infected plant (ZZD) with witches’-broom disease, pooled together and performed the modified 5’-RNA ligase-mediated (RLM)-RACE experiment to validate the cleavage sites. The 5’-RACE products revealed that 2 *SPL* geners and 1 *MYB* gene are indeed the targets of *Z*. *jujuba* miR156 and miR159, respectively (Fig 5). This is consistent with the results from other plants [33, 34], suggesting the functional conservation of miR156 and miR159.

## Discussion

In recent years, research on small RNA function and mechanism has become one of the hot spots in the life science. miRNAs as a new regulator, play important and diverse roles in multiple developmental and physiological processes, antiviral defense, responding to biotic, abiotic stresses etc. It has been indicated that miRNAs play an important role in plant-pathogen interactions, such as miR393, miR319, miR160, miR167, miR390, and miR408 [35]. A growing number of pathogen-responsive miRNAs have been identified [36]. To our best knowledge, the small RNAs of *Z*. *jujuba* have not been previously reported. In this study, a total of 85 conserved miRNA unique sequences belonging to 32 miRNA families and 24 novel miRNA unique sequences, including their complementary miRNA* strands were identified in two libraries derived from *Z*. *jujuba* wild type (ZZN) uninfected leaves and leaves infected (ZZD) with JWB disease. 40 target genes of 85 conserved miRNAs and 49 target genes of 24 novel miRNAs were predicted using computational analysis, and their functions were putatively assigned. We also identified differentially expressed miRNAs associated with JWB disease between ZZN and ZZD libraries. The targets of miR156 and miR159 were validated using the modified 5’-RACE method.

The regulatory mechanism of miRNAs involved in witches’-broom phytoplasma response is a complicated problem. Currently there are only two reported studies involving witches’-broom phytoplasma responsive miRNAs, which consist of the investigation of Mexian lime infected by *Candidatus* Phytoplasma aurantifolia [29] and mulberry infected by aster yellows phytoplasma [37]. Both these studies concluded that the differentially expressed miRNAs in healthy and phytoplasma infected plants involved in modulating multiple pathways such as hormonal, nutritional, and stress signaling pathways [29, 37]. They also concluded that these responsive sRNAs may work cooperatively in the response to phytoplasma infection and be responsible for some symptoms observed in the infected plants [38]. Compared to these two studies, among the 85 conserved witches’-broom phytoplasma responsive miRNAs identified in *Z*. *jujuba*, only 3 miRNA families, including miR156, miR172 and miR477, were also differentially expressed in *Candidatus* phytoplasma aurantifolia infected Mexican lime and aster yellows phytoplasma infected in mulberry. Therefore, the expression patterns of miRNAs responsive to the phytoplasma infection were diverse in different plants.

In this study, among the differentially expressed miRNAs, miR156 was the most up-regulated differentially miRNAs, suggesting that miR156 may play an important role in response to JWB. In both libraries, all the up-regulated miRNA sequences with a greater than 3.5 fold change were members of miR156 family. The SQUAMOSA PROMOTER BINDING PROTEIN LIKE (SPL) genes were the targets of miR156. Our results showed that miR156 was up-regulated in the infected sprig leaves, meanwhile miR172 was down-regulated. A previous study showed that the overexpression of miR156b in Arabidopsis increased axillary branching [39], which is similar to the symptom of the witches’-broom disease [38–40]. Furthermore, APETALA2 was regulated by miR172 through direct RNA cleavage or translational repression [36, 41]. In this study, we found the expression levels of miR172 was down-regulated, suggesting that the expression levels of APETALA2 gene was increased, which takes part in regulating flowering time and floral organ identity [41]. In addition, some *SPL* genes, such as *AtSPL9*, positively regulate the expression of miR172. This forms the miR156-*AtSPL9*-miR172 regulatory pathway [42, 43]. Therefore, the miR156-*SPL9*-miR172 regulatory pathway may be also conserved in response to phytoplasma infection. The expression changes of miR156 and miR172 might leads to the symptoms of the JWB diseases such as development of green leaf-like structures instead of flowers and sterility of flowers. MiR159 is the most abundant miRNAs in both libraries, which targets the mRNAs of MYB transcription factors. The expression level of miR159 was down-regulated in the ZZD library. It has been suggested that the overexpression of *MYB33* leads to rolled leaf and shorter petioles [44, 45]. Therefore, our results suggest that miR156, miR172 and miR159 play important roles in the responses to JWB diseases, which will provide the insight to elusive the molecular mechanisms of witches’-broom disease in *Z*. *jujuba*.

## Supporting Information

S1 Fig The predicted hairpin structures of all the novel miRNAs. (PDF)

S1 Table Primers used for qRT-PCR. (DOC)

S2 Table Primers used for validation of the miRNA cleavage of targets. (DOC)

S3 Table The expression profiling of miRNAs between ZZN and ZZD libraries. (DOC)
