# Supplementary material for: Genome-Wide Identification and Analysis of MicroRNAs Involved in Witches’-Broom Phytoplasma Response in Ziziphus jujuba
Source: PLoS One. 2016 Nov 8;11(11):e0166099. doi: 10.1371/journal.pone.0166099 (PMC5100886; doi:10.1371/journal.pone.0166099)

S1 Fig. The predicted hairpin structures of all the novel miRNAs.

zju-miRn1

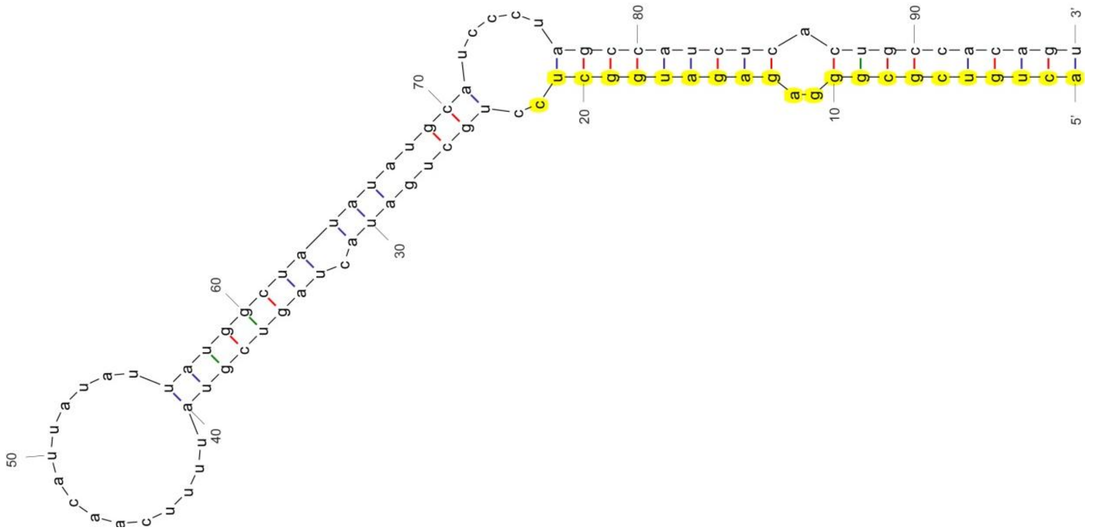

zju-miRn2

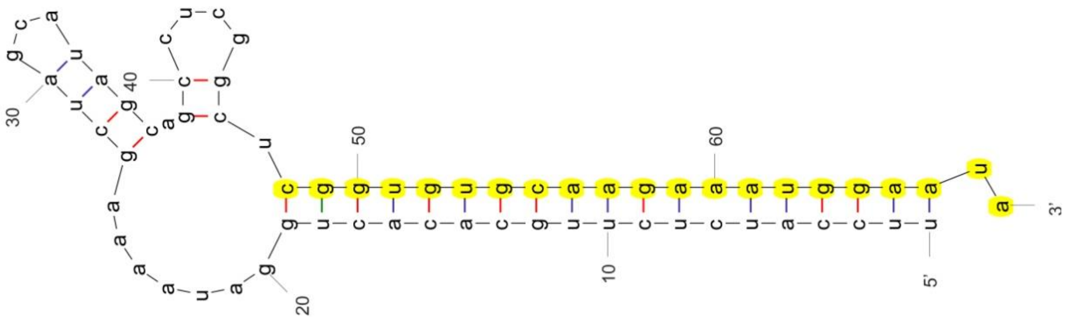

zju-miRn3

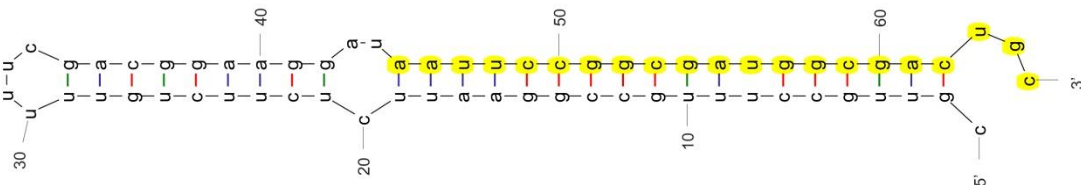

zju-miRn4

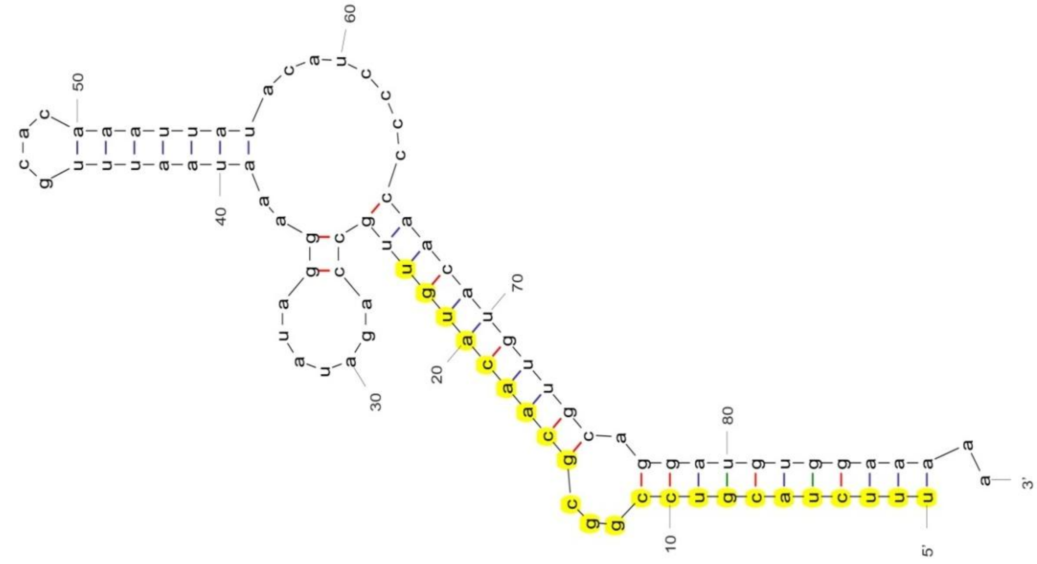

zju-miRn5

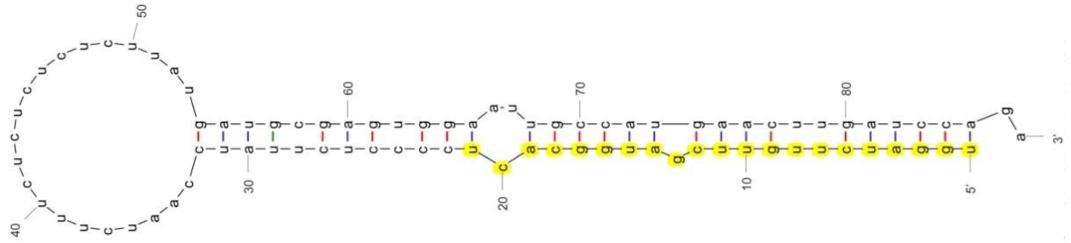

zju-miRn6

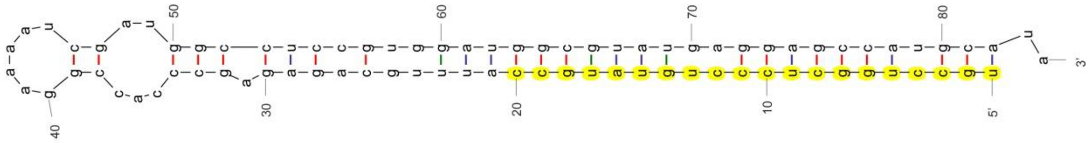

zju-miRn7

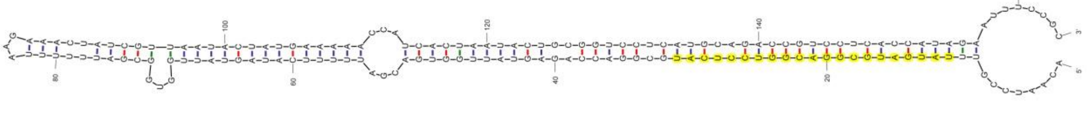

zju-miRn8

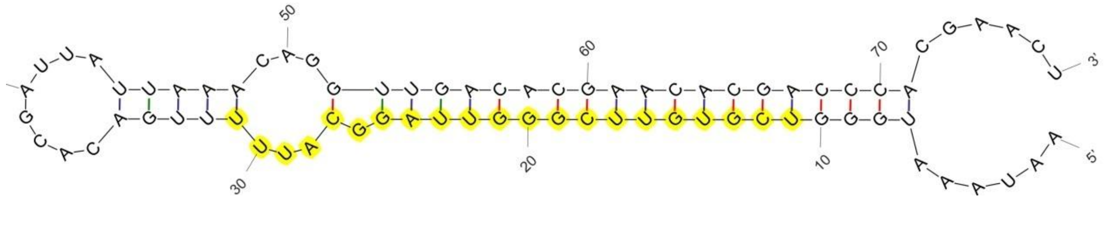

zju-miRn9

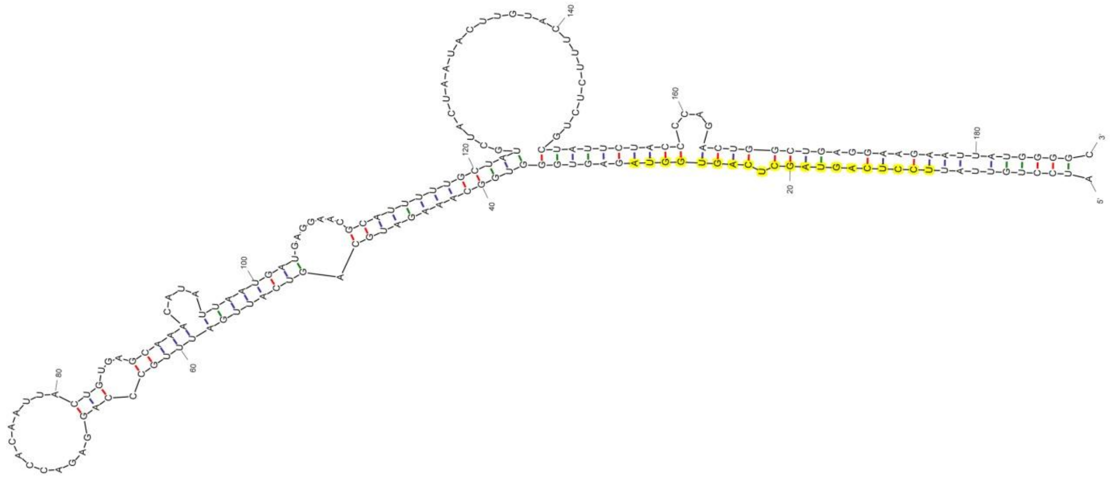

zju-miRn10

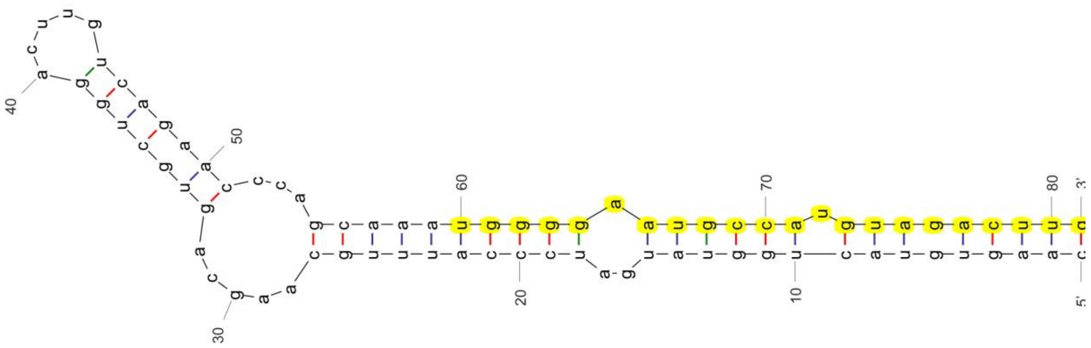

zju-miRn11

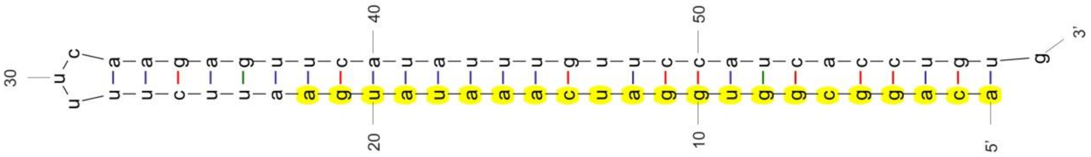

zju-miRn12

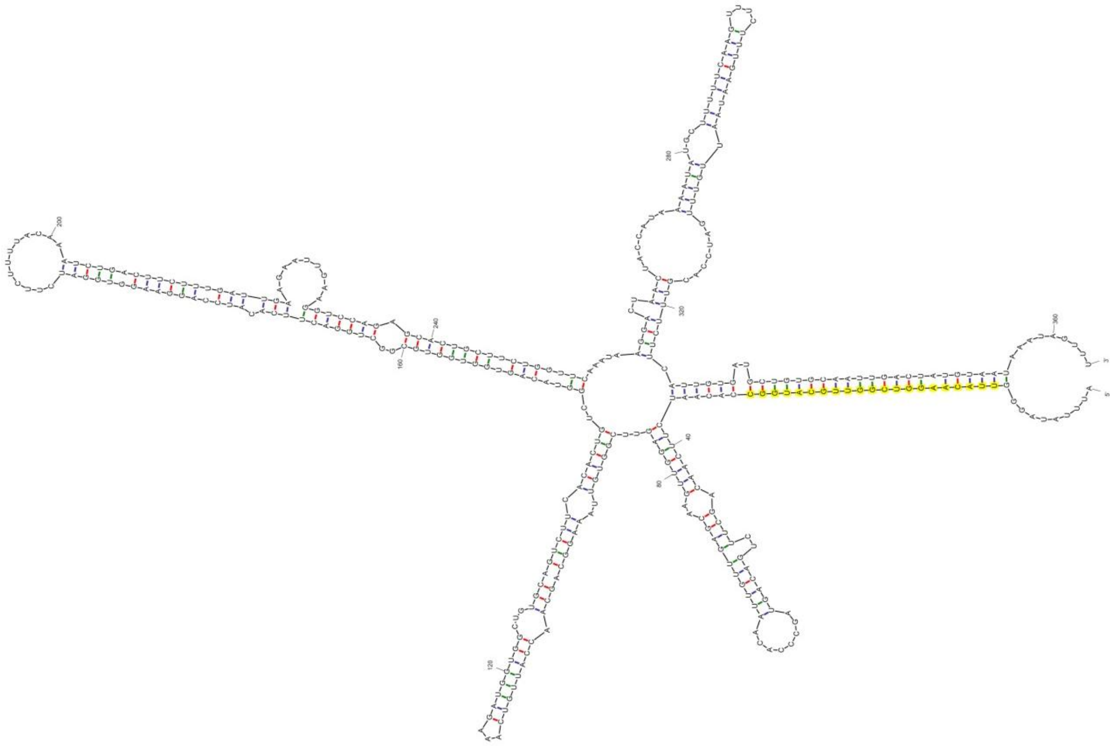

zju-miRn13

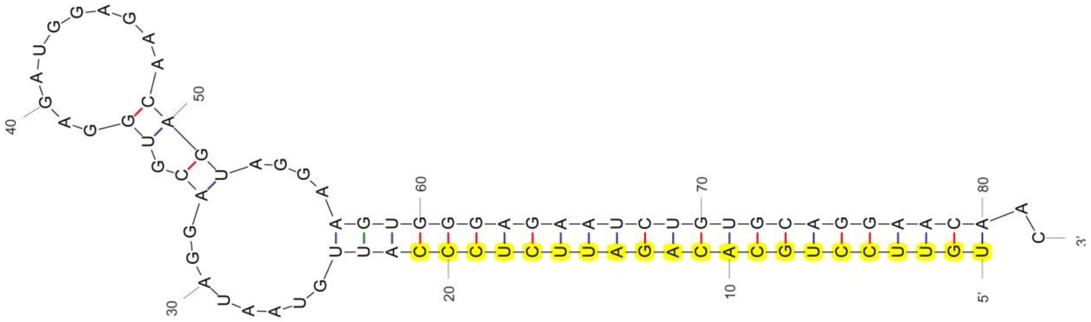

zju-miRn14

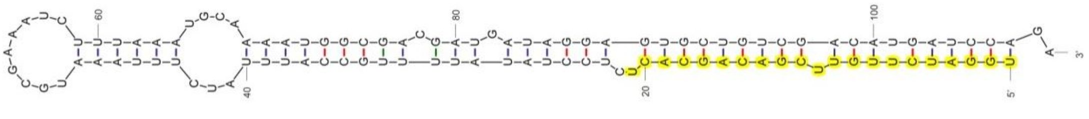

zju-miRn15

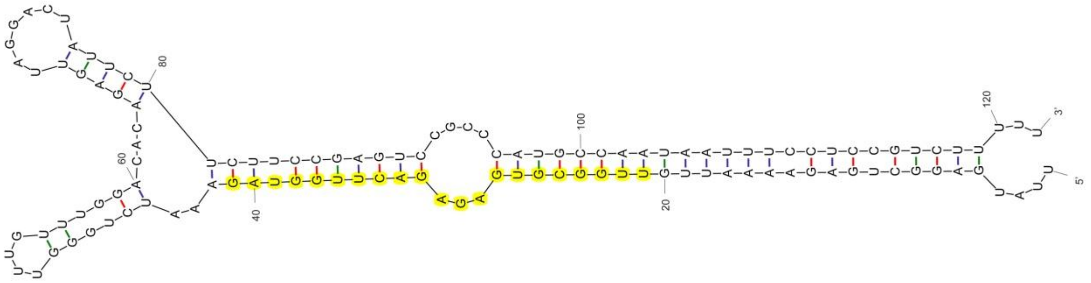

zju-miRn16

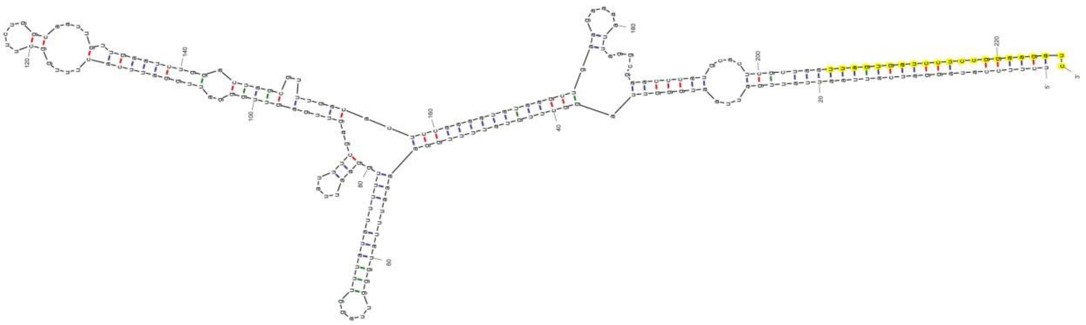

zju-miRn17

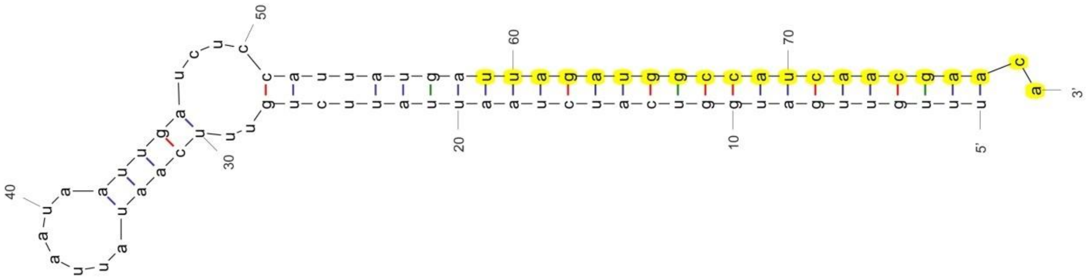

zju-miRn18

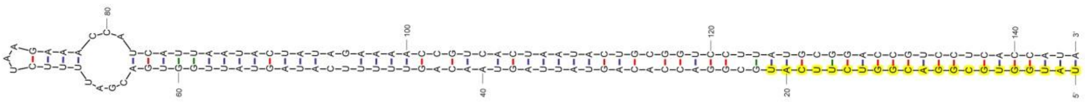

zju-miRn19

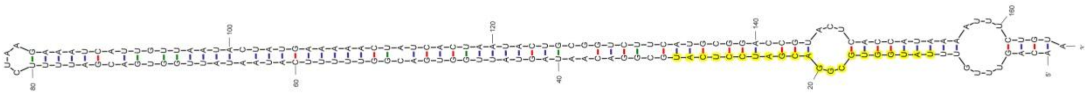

zju-miRn20

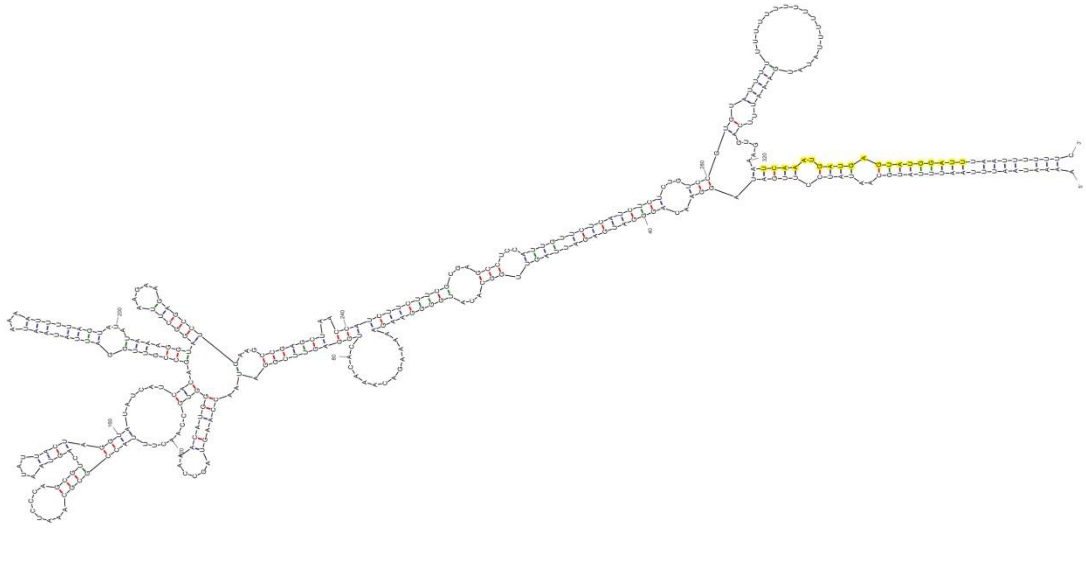

zju-miRn21

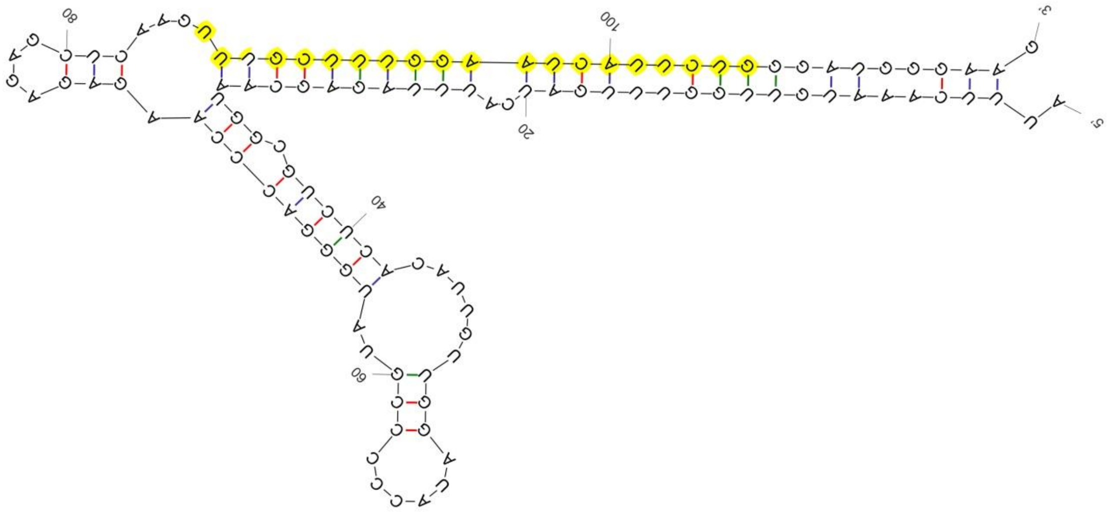

zju-miRn22

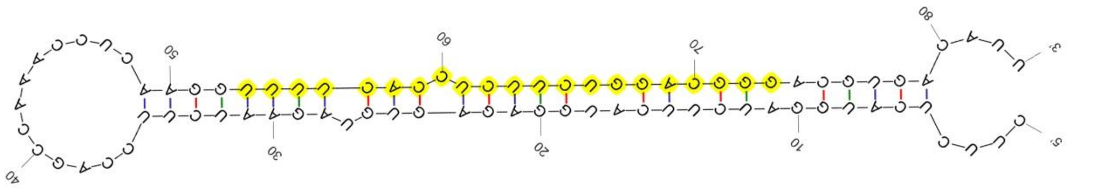

zju-miRn23

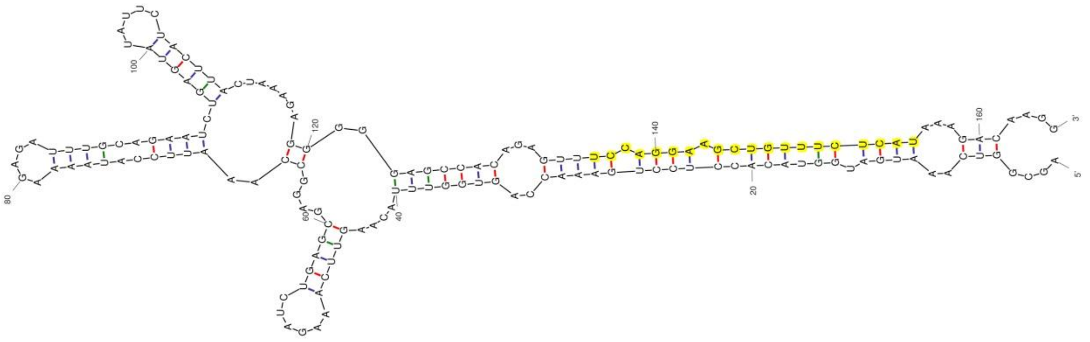

zju-miRn24

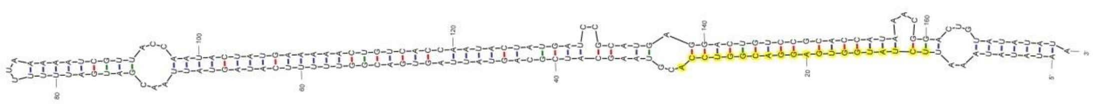

Supplement: S1 Fig — (PDF) [file pone.0166099.s001.pdf]
