# Supplementary material for: Genome-Wide Identification and Analysis of MicroRNAs Involved in Witches’-Broom Phytoplasma Response in Ziziphus jujuba
Source: PLoS One. 2016 Nov 8;11(11):e0166099. doi: 10.1371/journal.pone.0166099 (PMC5100886; doi:10.1371/journal.pone.0166099)
Supplement: S1 Table — (DOC) [file pone.0166099.s002.doc]

S1 Table. Primers used for qRT-PCR.

| **Gene name** | **Primer sequence (5' to 3')** |
| --- | --- |
| Reverse primer | GCGAGCACAGAATTAATACGAC |
| Poly(T) adapter | GCGAGCACAGAATTAATACGACTCACTATAGG(T)12VN |
| Zj5.8SrRNA | GCGATACTTGGTGTGAATTGCA |
| miR156a | TTGACAGAAGAGAGTGAGCAC |
| miR156c | TTGACAGAAGATAGAGAGCAC |
| miR156d | TGACAGAAGAGAGTGAGCAC |
| miR172 | AGAATCTTGATGATGCTGCAT |
| miR477 | ACTCTCCCTCAAGGGCTTCT |
| miR2111 | TAATCTGCATCCTGAGGTTTA |
