# Supplementary material for: Genome-Wide Identification and Analysis of MicroRNAs Involved in Witches’-Broom Phytoplasma Response in Ziziphus jujuba
Source: PLoS One. 2016 Nov 8;11(11):e0166099. doi: 10.1371/journal.pone.0166099 (PMC5100886; doi:10.1371/journal.pone.0166099)
Supplement: S2 Table — (DOC) [file pone.0166099.s003.doc]

S2 Table. Primers used for validation of the miRNA cleavage of targets.

| **miRNA name** | **Target gene** | **Primer Sequence (5' to 3')** |
| --- | --- | --- |
| miR156 | squamosa promoter-binding-like protein 12-like | Nesting: GGCTTGGGGAGCTCCAACATGTT |
|  |  | Nested: CAGGCCATCAGGACCCATTCTAA |
| miR156 | squamosa promoter-binding-like protein 13A-like | Nesting: GGCTTGGGGAGCTCCAACATGTT |
|  |  | Nested: GAGGCTTGTCTTTTCCATGATCA |
| miR159 | transcription factor GAMYB-like | Nesting: GCCATGGCACCAGGTGTAATACT |
|  |  | Nested: CAGCCGAATGATTCCTTCCACT |
| miR172 | transcription factor RAP2-7-like | Nesting: GTTCTGTGCTTGGGTATTGTA |
|  |  | Nested: CCGAAACAACATCTCTACAGAGT |
| miR477 | DELLA protein GAI1 | Nesting: CCTCTGTTTCCAACGCCGGTTAT |
|  |  | Nested: GCGTTGAAGTGGTACTCTTCCTCT |
| miR2111 | Cell division protein ftsZ | Nesting: GCTGCATACTACTTGCTGCTGT |
|  |  | Nested: GGTATTTGTTAGCTGTCGAGCGCA |
