# Supplementary material for: Genome-Wide Identification and Analysis of MicroRNAs Involved in Witches’-Broom Phytoplasma Response in Ziziphus jujuba
Source: PLoS One. 2016 Nov 8;11(11):e0166099. doi: 10.1371/journal.pone.0166099 (PMC5100886; doi:10.1371/journal.pone.0166099)
Supplement: S3 Table — (DOC) [file pone.0166099.s004.doc]

S3 Table. The expression profiling of miRNAs between ZZN and ZZD libraries.

| **miRNA** | **normalized read count** | | **log2.Fold_change** | **regulate** |
| --- | --- | --- | --- | --- |
|  | **ZZN** | **ZZD** |  |  |
| miR1515 | 11.7 | 4.5 | -1.4 | down-regulated |
| miR156a | 23.3 | 423.3 | 4.2 | up-regulated |
| miR156b | 18.2 | 201.8 | 3.5 | up-regulated |
| miR156c | 13.1 | 264.6 | 4.3 | up-regulated |
| miR156d | 16.0 | 1049.3 | 6.0 | up-regulated |
| miR156e | 2.9 | 14.3 | 2.3 | up-regulated |
| miR156f | 21.9 | 35.9 | 0.7 | up-regulated |
| miR156h | 2.2 | 25.1 | 3.5 | up-regulated |
| miR159a | 8.0 | 0.9 | -3.2 | down-regulated |
| miR159b | 50131.4 | 23937.6 | -1.1 | down-regulated |
| miR159c | 52649.2 | 36321.1 | -0.5 | down-regulated |
| miR159d | 1125.3 | 649.3 | -0.8 | down-regulated |
| miR159e | 229.4 | 2022.4 | 3.1 | up-regulated |
| miR160a | 61.9 | 91.5 | 0.6 | up-regulated |
| miR160b | 26.9 | 16.1 | -0.7 | down-regulated |
| miR160c | 45.9 | 54.7 | 0.3 | up-regulated |
| miR160d | 56.8 | 47.5 | -0.3 | down-regulated |
| miR162a | 4539.7 | 2736.3 | -0.7 | down-regulated |
| miR162b | 18.9 | 8.1 | -1.2 | down-regulated |
| miR164a | 32.0 | 13.5 | -1.3 | down-regulated |
| miR164b | 98.3 | 123.8 | 0.3 | up-regulated |
| miR166a | 726.9 | 275.3 | -1.4 | down-regulated |
| miR166b | 21.9 | 19.7 | -0.1 | down-regulated |
| miR166c | 39579.2 | 21283.8 | -0.9 | down-regulated |
| miR166d | 247.6 | 227.8 | -0.1 | down-regulated |
| miR166e | 199.6 | 174.9 | -0.2 | down-regulated |
| miR166f | 251.3 | 243.0 | 0.0 | down-regulated |
| miR166g | 42.2 | 32.3 | -0.4 | down-regulated |
| miR167a | 309.5 | 153.4 | -1.0 | down-regulated |
| miR167b | 663.5 | 312.1 | -1.1 | down-regulated |
| miR167c | 11045.2 | 6133.5 | -0.8 | down-regulated |
| miR167d | 377.3 | 261.0 | -0.5 | down-regulated |
| miR167e | 16.0 | 10.8 | -0.6 | down-regulated |
| miR167f | 1876.9 | 890.6 | -1.1 | down-regulated |
| miR167g | 2016.0 | 717.5 | -1.5 | down-regulated |
| miR167h | 9799.0 | 6370.3 | -0.6 | down-regulated |
| miR168a | 793.9 | 522.0 | -0.6 | down-regulated |
| miR168b | 56.8 | 32.3 | -0.8 | down-regulated |
| miR168c | 62.6 | 39.5 | -0.7 | down-regulated |
| miR170a | 102.7 | 376.7 | 1.9 | up-regulated |
| miR171a | 8.0 | 31.4 | 2.0 | up-regulated |
| miR171b | 33.5 | 82.5 | 1.3 | up-regulated |
| miR172 | 188.6 | 4.5 | -5.4 | down-regulated |
| miR2111a | 15.3 | 0.9 | -4.1 | down-regulated |
| miR2950a | 137.7 | 9.9 | -3.8 | down-regulated |
| miR319a | 33.5 | 310.3 | 3.2 | up-regulated |
| miR319b | 26.9 | 42.2 | 0.6 | up-regulated |
| miR319c | 21.1 | 16.1 | -0.4 | down-regulated |
| miR319d | 40.1 | 50.2 | 0.3 | up-regulated |
| miR319e | 285.5 | 578.5 | 1.0 | up-regulated |
| miR319f | 60.5 | 52.0 | -0.2 | down-regulated |
| miR319g | 319.0 | 429.6 | 0.4 | up-regulated |
| miR384 | 37.9 | 73.5 | 1.0 | up-regulated |
| miR390 | 5.1 | 10.8 | 1.1 | up-regulated |
| miR391a | 797.5 | 259.2 | -1.6 | down-regulated |
| miR391b | 40.8 | 26.0 | -0.6 | down-regulated |
| miR394 | 37.9 | 73.5 | 1.0 | up-regulated |
| miR395a | 4.4 | 26.0 | 2.6 | up-regulated |
| miR395b | 16.0 | 67.3 | 2.1 | up-regulated |
| miR396a | 45.9 | 42.2 | -0.1 | down-regulated |
| miR396b | 44.4 | 28.7 | -0.6 | down-regulated |
| miR396c | 26.9 | 17.9 | -0.6 | down-regulated |
| miR396d | 10603.8 | 6274.3 | -0.8 | down-regulated |
| miR396e | 28954.3 | 28643.3 | 0.0 | down-regulated |
| miR396f | 1960.7 | 1462.7 | -0.4 | down-regulated |
| miR396g | 26.2 | 29.6 | 0.2 | up-regulated |
| miR396h | 13.8 | 23.3 | 0.8 | up-regulated |
| miR397 | 38.6 | 20.6 | -0.9 | down-regulated |
| miR398a | 232.3 | 60.1 | -2.0 | down-regulated |
| miR398b | 8306.7 | 4364.9 | -0.9 | down-regulated |
| miR398c | 442.1 | 132.7 | -1.7 | down-regulated |
| miR399 | 24.0 | 1.8 | -3.7 | down-regulated |
| miR403 | 148.6 | 147.1 | 0.0 | down-regulated |
| miR408a | 1304.4 | 601.8 | -1.1 | down-regulated |
| miR408b | 635.1 | 216.1 | -1.6 | down-regulated |
| miR408c | 592.9 | 230.5 | -1.4 | down-regulated |
| miR477 | 56.8 | 3.6 | -4.0 | down-regulated |
| miR482a | 21.1 | 26.0 | 0.3 | up-regulated |
| miR482b | 8.0 | 12.6 | 0.6 | up-regulated |
| miR529a | 1111.4 | 3080.6 | 1.5 | up-regulated |
| miR529b | 51.0 | 114.8 | 1.2 | up-regulated |
| miR530 | 16.8 | 15.2 | -0.1 | down-regulated |
| miR6478 | 609.6 | 259.2 | -1.2 | down-regulated |
| miR858a | 375.8 | 111.2 | -1.8 | down-regulated |
| miR858b | 61.9 | 10.8 | -2.5 | down-regulated |
| zju-miRn1 | 33.5 | 9.9 | -1.8 | up-regulated |
| zju-miRn2 | 11.7 | 0.9 | -3.7 | up-regulated |
| zju-miRn3 | 14.6 | 3.6 | -2.0 | up-regulated |
| zju-miRn4 | 5.1 | 3.6 | -0.5 | up-regulated |
| zju-miRn5 | 9148.6 | 6009.7 | -0.6 | up-regulated |
| zju-miRn6 | 0.7 | 0.9 | 0.3 | down-regulated |
| zju-miRn7 | 5.8 | 9.0 | 0.6 | down-regulated |
| zju-miRn8 | 39.3 | 9.0 | -2.1 | up-regulated |
| zju-miRn9 | 56.1 | 49.3 | -0.2 | up-regulated |
| zju-miRn10 | 7.3 | 2.7 | -1.4 | up-regulated |
| zju-miRn11 | 453.8 | 348.0 | -0.4 | up-regulated |
| zju-miRn12 | 5.8 | 9.0 | 0.6 | down-regulated |
| zju-miRn13 | 67.0 | 49.3 | -0.4 | up-regulated |
| zju-miRn14 | 344.5 | 884.3 | 1.4 | down-regulated |
| zju-miRn15 | 34.2 | 75.3 | 1.1 | down-regulated |
| zju-miRn16 | 356.9 | 49.3 | -2.9 | up-regulated |
| zju-miRn17 | 161.7 | 43.9 | -1.9 | up-regulated |
| zju-miRn18 | 14.6 | 10.8 | -0.4 | up-regulated |
| zju-miRn19 | 21.9 | 16.1 | -0.4 | up-regulated |
| zju-miRn20 | 35.7 | 77.1 | 1.1 | down-regulated |
| zju-miRn21 | 10.9 | 26.0 | 1.3 | down-regulated |
| zju-miRn22 | 2.9 | 10.8 | 1.9 | down-regulated |
| zju-miRn23 | 0.0 | 10.8 | - | up-regulated |
| zju-miRn24 | 0.0 | 43.9 | - | up-regulated |
